# Exogenous Administration of Delta-9-Tetrahydrocannabinol Affects Adult Hippocampal Neurotransmission in Female Wistar Rats

**DOI:** 10.3390/ijms26136144

**Published:** 2025-06-26

**Authors:** Ana M. Neves, Sandra Leal, Bruno M. Fonseca, Susana I. Sá

**Affiliations:** 1 Department of Biomedicine, Unit of Anatomy, Faculty of Medicine of the University of Porto (FMUP), Alameda Professor Hernani Monteiro, 4200-319 Porto, Portugal; up201906545@up.pt; 2 Associate Laboratory i4HB—Institute for Health and Bioeconomy, University Institute of Health Sciences—CESPU, 4585-116 Gandra, Portugal; sandra.leal@iucs.cespu.pt; 3 UCIBIO—Applied Molecular Biosciences Unit, University Institute of Health Sciences—CESPU (1HTOXRUN, IUCS-CESPU), 4585-116 Gandra, Portugal; 4 Associate Laboratory i4HB—Institute for Health and Bioeconomy, Faculty of Pharmacy of the University of Porto (FFUP), R. Jorge de Viterbo Ferreira 228, 4050-313 Porto, Portugal; brunofonseca@ff.up.pt; 5 RISE—HEALTH, Department of Biomedicine, Unit of Anatomy, Faculty of Medicine of the University of Porto (FMUP), Alameda Professor Hernani Monteiro, 4200-319 Porto, Portugal

**Keywords:** *Cannabis*, THC, cannabinoid receptors, hippocampal formation, estradiol, female rats

## Abstract

Delta-9-tetrahydrocannabinol (THC) is a psychoactive element of *Cannabis sativa* and affects the human cannabinoid system through its receptors, CB1R and CB2R. CB1R was found in several brain areas, including the hippocampal formation (HF), and it is responsible for most THC side effects. We investigated THC’s effects in the HF of female Wistar rats to assess changes in its neurotransmission. Female Wister rats (*n* = 20) were gonadectomized under anesthesia at 8 weeks old. Afterwards, they received estradiol benzoate (EB) and/or THC. Immunohistochemistry was performed to assess the expression of the cholinergic receptor alpha 7 subunit (CHRNA7), the vesicular acetylcholine transporter (VAChT), the vesicular glutamate transporter (VGLUT), the gamma-aminobutyric acid type A receptor (GABRA), the CB1 receptor, and estradiol receptor alpha (EBα). In the HF, the expression of CHRNA7 was increased by EB and by THC in the Oil groups but decreased by THC in the EB groups. The same is true for VGLUT expression in the DG and hilum and for GABRA expression in the hilum. The expression of VAChT and CB1 is reduced by EB, while the concomitant administration of THC increases it. GAD expression is reduced by EB administration in CA1, CA3, and DG. Our results may help with decision-making regarding the prescription of low doses of THC as a therapeutical approach.

## 1. Introduction

### 1.1. Overview of Delta-9-Tetrahydrocannabinol and Cannabis

Delta-9-tetrahydrocannabinol (THC) is the most potent psychoactive element of *Cannabis sativa*, a plant whose extract has been used for a variety of purposes for centuries, including medical, recreational, and religious purposes [1]. The use of *Cannabis* was decriminalized in Portugal in 2000 (Lei no.30/2000), and its medical use was legalized in 2018 (Lei no. 33/2018). *Cannabis* is medically approved for chronic pain, chemotherapy-induced nausea and vomiting, the stimulation of appetite, anxiety disorders, Parkinson’s disease, and epilepsy, among others [2,3].

The effects of *Cannabis* arise from the action of its psychoactive elements in the human cannabinoid system. This system is activated by cannabinoids, which can be either exogenous (like *Cannabis*’ THC—the only of its elements with psychotropic and hallucinogenic properties) [4] or endogenous. The cannabinoid transmission occurs in a retrograde way, in opposition to cholinergic, glutamatergic, and gamma-aminobutyric acid (GABA)-ergic transmission. Retrograde signaling involves the release of a messenger by postsynaptic cells, which travels through the synaptic cleft to the receptors on presynaptic cells—it is a more precise, rapid, and efficient way to regulate transmission on synapses [5].

The effects of cannabinoids arise from their action on cannabinoid receptors, CB1 and CB2 [3,6]. CB1 is mostly found in the brain [6], namely in areas involved in mood, cognitive functions, and motor control, including the hippocampal formation (HF) [1,7]. CB2 is mostly found in the periphery [6], and it has anti-inflammatory, regenerative, and antioxidant properties [1,4]. THC activates both, but mainly CB1 [3,6], where it can act both as an agonist and as an antagonist, depending on the system it is acting on [6]. THC is responsible for the changes in perception, mood, and cognition that are frequently sensed in *Cannabis* users [4].

The action of the psychoactive elements of *Cannabis* on CB1 in the brain is also the cause of its side effects [3]. They range from dry mouth, tachycardia or bradycardia, sedation, nausea, vomiting, and gastrointestinal distress to confusion, disorientation, and psychosis [1,2]. It has also been shown that THC causes deficits in long-term synaptic plasticity, impairing learning, memory, and attention [7,8,9].

### 1.2. Hippocampal Formation and Cannabis

**Figure 1:**
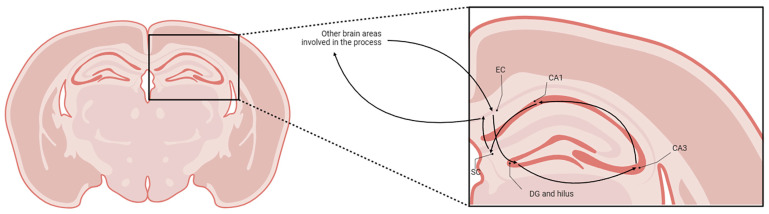
Schematic representation of the trisynaptic memory pathway (created using BioRender.com).

The brain HF comprises the *cornu ammonis* (CA—divided into CA1, CA2, and CA3), the dentate gyrus (DG—whose internal layer is named the hilus), the subicular complex (SC), and the entorhinal cortex (EC) [10]. The HF is involved in learning, memory, spatial navigation, and emotional behavior [11]. Memory seems to be one of the most critical functions, and its circuit within the HF is complex: the trisynaptic pathway proceeds as illustrated in Figure 1 [12].

While executing its functions, the HF interacts with many neurotransmitter systems [11]. Regarding the cholinergic system, acetylcholine favors memory encoding to the detriment of memory consolidation, acting through the activation of CA1 circuits that carry extrinsic information involved in the coding process while inhibiting CA3 intrinsic pathways involved in memory consolidation, playing an important role in the types of memory that are dependent on the hippocampus, namely episodic and spatial memory [13]. Regarding the glutamatergic system, glutamate plays an important role in memory, especially by acting in CA3 cells, where it seems to reduce inhibition and facilitate excitation during the execution of memory functions, while diminishing DG signaling to increase the sensitivity of CA3, which plays an important role in synaptic plasticity [12]. Regarding the GABAergic system, GABA plays an important role in the neuronal plasticity of the HF circuits involved in memory and learning, although the mechanism is still unknown [14].

Studies suggest that memory impairments associated with *Cannabis* use arise from the actions of its psychoactive elements in the CB1 receptors abundantly present in the HF [7]. THC affects the HF’s neurotransmitter release through this receptor [15]. Neurochemical studies also revealed that THC is related to a decrease in the brain release and turnover of acetylcholine, which may explain some of the side effects of *Cannabis* use [16]. There are studies that demonstrate that THC inhibits both glutamatergic and GABAergic transmission, with higher potential to inhibit the latter [7,17], possibly resulting in deficits in long-term synaptic plasticity and memory [15].

### 1.3. Sexual Dimorphism in Cannabis’ Effects

Men and women use and are affected by *Cannabis* differently. It is typically consumed more by men, although in recent years the consumption by women has been increasing, narrowing the gap between the sexes [2]. Some human studies suggest that women users may be more sensitive to THC’s effects and develop greater tolerance for the drug [2,18]. Animal studies suggest that THC seems to have more analgesic effects but more anxiogenic effects in females, including tachycardia [2]. A few animal studies in females suggest that estradiol is somewhat responsible for the increased sensitivity to THC’s analgesic effects, although the mechanism is unknown [2]. It is also known that THC can modulate estradiol binding to its receptor [19,20].

Some studies also describe a greater improvement in appetite in female patients undergoing chemotherapy when compared to male patients [2]. In addition, THC seems to have more pronounced side effects on the female sex, particularly alterations in movement, nausea, and reproductive health (such as impaired fertility), though more studies are needed in this area [2,21].

### 1.4. Aim of the Study

In this study, we aim to investigate the possible changes in neurotransmission systems in the female rat HF after THC exposure and to correlate these changes with the effects that are known to be provoked by THC in *Cannabis* users. We studied the three systems highlighted previously: the cholinergic system, which was studied through determining the possible effects on the expression of the cholinergic receptor alpha 7 subunit (CHRNA7) and the vesicular acetylcholine transporter (VAChT), and the glutamatergic and GABAergic systems, which were studied through analyzing the changes in the vesicular glutamate transporter (VGLUT), the gamma-aminobutyric acid type A receptor (GABRA), and glutamic acid decarboxylase (GAD) expression. We also studied the expression of two other proteins of interest: CB1 and estradiol receptor alpha (EBα). With an increase in *Cannabis* consumption by women and given both the influence of estradiol and a lack of studies on this drug’s effects on this sex, we chose to focus our study on female rats. Our results can contribute to explaining the pathway through which THC affects human behavior and, hopefully, inform decisions about its use in clinical and daily practice.

## 2. Results

When performing the results analysis, we chose to combine the analyses of the results of all the areas of the HF if the variation in the expression of the protein was the same in every HF area studied. In these cases, the results are presented as HF. The results are presented as the mean ± standard error of the mean (SEM), with statistical significance set at *p* ≤ 0.05.

### 2.1. Effects of THC Administration on the Expression of CHRNA7 in HF Areas

**Figure 2:**
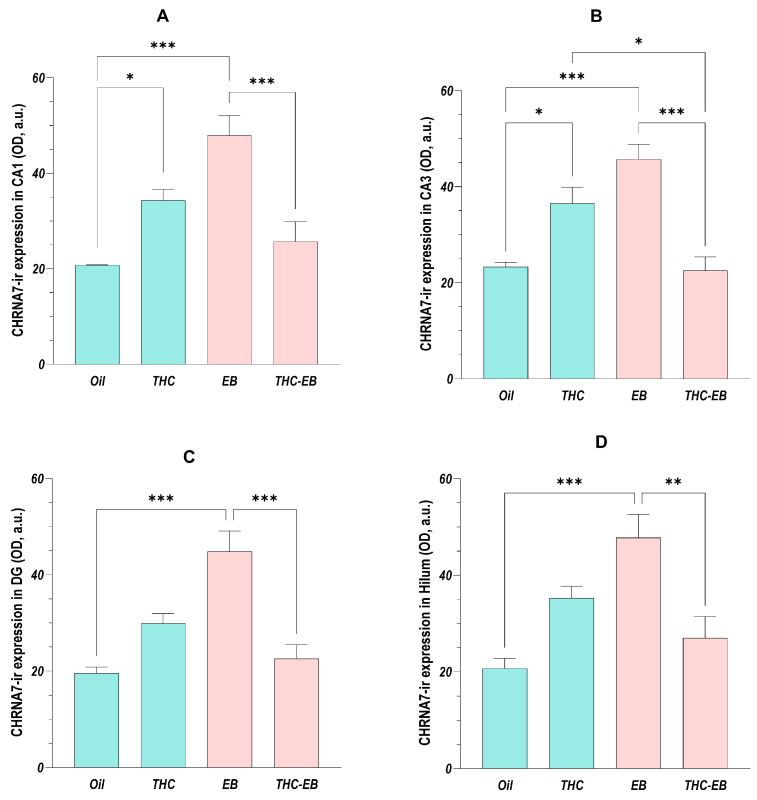
CHRNA7 expression variation in HF in groups treated with the vehicle (Oil), delta-9-tetrahydrocannabinol (THC), estradiol benzoate (EB), and THC-EB, divided by areas: CA1 (**A**), CA3 (**B**), dentate gyrus (DG; (**C**)), and hilum (**D**). Columns represent the means ± SEM. Tukey’s Post Hoc tests: *** *p* < 0.001, ** *p* < 0.01, * *p* < 0.05, comparing paired groups.

The ANOVA analysis showed that the administration of THC affects the HF areas in different ways. The administration of EB increased the expression of the receptor in both CA1 [F (3, 16) = 14.11; *p* < 0.001] and CA3 [F (3, 16) = 16.54; *p* < 0.001] two-fold; however, the simultaneous administration of THC decreased its expression to half (group THC-EB) but increased its expression two-fold in animals that did not receive EB (group THC). The expression in the THC-EB group was approximately 60% of the expression in the THC group in CA3. Regarding the DG [F (3, 16) = 15.68; *p* < 0.001] and hilum [F (3, 16) = 10.23; *p* < 0.001], the administration of EB in animals that did not receive THC increased the receptor’s expression twice but decreased its expression by 40 to 50% when administered concomitantly with THC (Figure 2).

### 2.2. Effects of THC Administration on the Expression of VAChT in the HF

**Figure 3:**
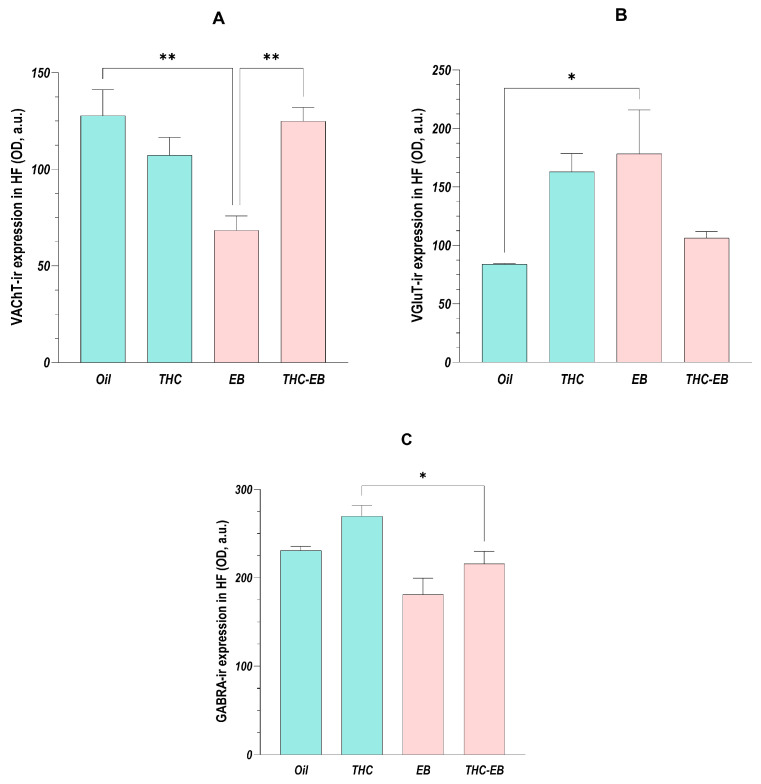
VAChT (**A**), VGLUT (**B**), and GABRA (**C**) expression variation in HF in groups treated with the vehicle (Oil), delta-9-tetrahydrocannabinol (THC), estradiol benzoate (EB), and THC-EB. All areas analyzed as one. Columns represent the means ± SEM. Tukey’s Post Hoc tests: ** *p* < 0.01, * *p* < 0.05, comparing paired groups.

The ANOVA analysis showed that the administration of THC affects every HF region in the same way regarding VAChT expression, so, as stated, we analyzed the data of the different areas together [F (3, 16) = 8.12; *p* < 0.01]. Post-hoc analysis showed that the administration of EB reduced the transporter’s expression by 40 to 50% in all the HF areas and that the concomitant administration of THC (group THC-EB) increased it by approximately two-fold in every area (Figure 3A).

### 2.3. Effects of THC Administration on the Expression of VGLUT in the HF

The ANOVA analysis showed that the administration of THC affected every HF area in the same way regarding VGLUT expression [F (3, 16) = 4.84; *p* = 0.01]; however, post-hoc analysis showed that only the DG and hilum had a variation in expression. Indeed, the administration of EB in the Oil group increased the VGLUT expression to double, though this effect was eliminated when THC was administered simultaneously (Figure 3B).

### 2.4. Effects of THC Administration on the Expression of GABRA in the HF

The ANOVA analysis showed that the administration of THC affected every HF area in the same way regarding GABRA expression [F (3, 16) = 7.63; *p* < 0.01]; however, post-hoc analysis showed that only the hilum presented a variation in the receptor expression. In this area, the animals of the THC-EB group presented less expression than the animals of the THC group (Figure 3C).

### 2.5. Effects of THC Administration on the Expression of GAD in HF Areas

**Figure 4:**
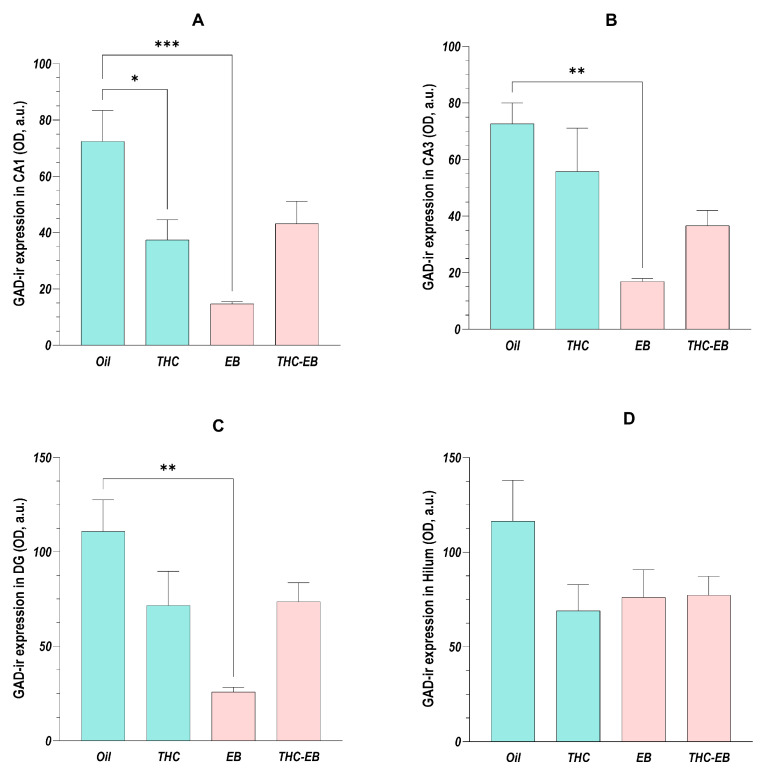
GAD expression variation in HF in groups treated with the vehicle (Oil), delta-9-tetrahydrocannabinol (THC), estradiol benzoate (EB), and THC-EB, divided by areas: CA1 (**A**), CA3 (**B**), dentate gyrus (DG, (**C**)), and hilum (**D**). Columns represent means ± SEM. Tukey’s Post Hoc tests: *** *p* < 0.001, ** *p* < 0.01, * *p* < 0.05, comparing paired groups.

The ANOVA analysis showed that the administration of THC only affected GAD expression in CA1 [F (3, 16) = 9.52; *p* < 0.001], CA3 [F (3, 16) = 7.27; *p* < 0.01], and DG [F (3, 16) = 6.84; *p* < 0.01], having no effect on GAD expression in the hilum [F (3, 16) = 1.90; *p* = 0.17]. Post-hoc analysis showed that, in the three areas, EB administration reduced GAD expression by 80%; however, THC administration in the rats that received EB (group THC-EB) eliminated this variation. THC administration in the rats that did not receive EB reduced the GAD expression by 50% in CA1 (Figure 4A).

### 2.6. Effects of THC Administration on the Expression of CB1 in the HF

**Figure 5:**
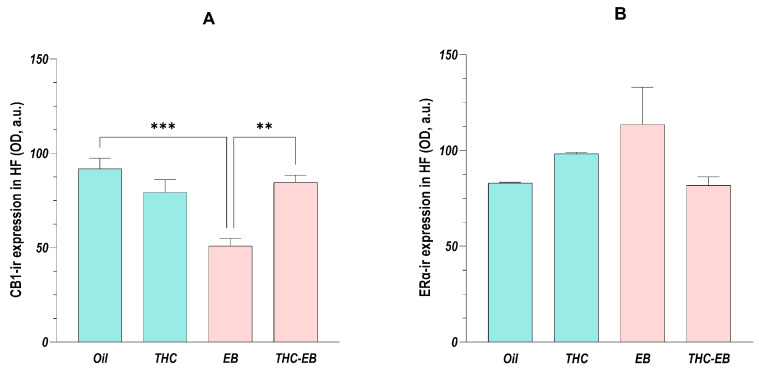
CB1 (**A**) and ERα (**B**) expression variation in HF in groups treated with the vehicle (Oil), delta-9-tetrahydrocannabinol (THC), estradiol benzoate (EB), and THC-EB. All areas analyzed as one. Columns represent means ± SEM. Tukey’s Post Hoc tests: *** *p* < 0.001, ** *p* < 0.01, comparing paired groups.

The ANOVA showed that the administration of THC affected every HF area in the same way regarding CB1 variation, so, as stated above, we analyzed the data of the different areas together [F (3, 16) = 12.07; *p* < 0.001]. Post-hoc analysis showed that the animals in group EB had a 50% reduction in CB1 expression and that THC administration in those animals (group THC-EB) increased the receptor’s expression by about 1.5-fold (Figure 5A).

### 2.7. Effects of THC Administration on the Expression of ERα in the HF

The ANOVA analysis showed that the administration of THC did not affect the HF areas regarding ERα expression, so, as stated, we analyzed the data of the different areas together [F (3, 16) = 2.25; *p* = 0.12]. Post-hoc analysis also showed no differences between the different groups (Figure 5B).

## 3. Discussion

Our study aimed to analyze the effects of THC on HF neurotransmission while additionally observing its relationship with EB, since the study used female rats. Our data suggests that understanding the relationship between these two agents is important to understand THC effects on female brain function, particularly in the three neurotransmission circuits we highlighted above.

### 3.1. The Cholinergic System

As stated above, we used both CHRNA7 and VAChT to study the cholinergic system. According to the literature, THC does have an impact on the cholinergic system, and our results corroborate those of previous studies.

The present results show that both THC and EB induced an increase in CHRNA7 expression when administered alone. However, when administered together, their effects invalidated each other, with CHRNA7 expression returning to values observed when neither of them was administered. This suggests that, in the female brain, THC can modulate CHRNA7 expression. Previous research in both rats and humans suggested that, in the medical field, the increase in CHRNA7 by THC might indicate the application of cannabinoids in the treatment of central nervous system (CNS)-related diseases where the receptor is known to play an important role (i.e., depression, epilepsy, autism, schizophrenia, Alzheimer’s disease, Parkinson’s disease, and stroke), particularly through anti-inflammatory effects and their impact on memory and learning [22,23]. Our study also showed that CHRNA7 modulation by THC is impacted by the female hormonal milieu and, therefore, the female cycle phase must be taken into consideration when considering cannabinoid therapy in women—doses of THC should be considered carefully if the therapy is to be given to fertile woman (prior to menopause), since it seems that the drug’s effects may be decreased by increased estradiol levels.

Although in a different direction, the results convey a similar pattern when we analyze VAChT. It is known that VAChT is essential for acetylcholine release [24]; however, data in the literature are rather poor regarding the relationship between THC and VAChT. In combining the results of CHRNA7 and VAChT, we hypothesize that a reduction in VAChT after THC and EB administration may indicate an increased turnover of VAChT to deliver higher rates of acetylcholine rather than a decrease in its production (and subsequently in acetylcholine release). In fact, substantial evidence implicates CHRNA7 and VAChT in neurobiological pathways critical for cognitive functions, including attention, learning, memory, and cognitive flexibility [25,26,27,28]. Increased expression of CHRNA7 has been positively associated with neuroprotection, the activation of anti-inflammatory pathways, and the regulation of hippocampal synaptic plasticity [29,30,31]. In addition, VAChT levels influence the rhythm strength of hippocampal cells, affecting recognition memory and contextual decision-making [32]. Moreover, estrogen has been shown to enhance cognitive performance by increasing the activity of the cholinergic system [27,28]. Nonetheless, altered cholinergic signaling in the hippocampus has been linked to anxiety-related behaviors, reduced exploration, lower emotional responsiveness, decreased locomotion, and impaired cognitive flexibility [26,33]. Our previous study showed that THC modulates social and sexual behaviors depending on the gonadal hormonal milieu [34], suggesting THC’s influence on decision-making. Similarly, *Cannabis* use in adolescents and young female adults is linked to increased risky sexual behavior, reflecting cross-species effects on arousal and motivation [35]. Consistent with this, THC exposure in HIV-1 transgenic rats showed worsened risk-based decision-making, despite enhancing learning performance [36].

### 3.2. The Glutamatergic and GABAergic Systems

The glutamatergic and GABAergic systems were studied through VGLUT, GABRA, and GAD expression in the HF. Since these systems’ regulations have a very strong relationship to ensure neurotransmission balance, their results will be discussed together. We did not observe a significant effect of THC on the expression of any of the said proteins. However, we do know that *Cannabis* consumption has an impact on these systems [15], as described previously. It is also known that these systems are very strictly regulated to ensure balance between excitation and inhibition on the hippocampus [37,38]; so, we hypothesize that although THC probably has momentary effects on these systems, the regulation process returns the expression of these proteins to homeostasis, blocking significant quantifiable variation by THC. Furthermore, it has been described that THC’s effects on these systems depend on CB1 receptor activation [15], which we did not observed. In this way, and despite the strangeness of such a result, this could explain the absence of the THC effect detected on these systems.

The absence of effect regarding these proteins might also be related to a small sample size. Perhaps with larger groups or a different time frame, we would be able to observe significant effects of THC on the expression of these proteins in the HF.

### 3.3. CB1 and ERα Receptors

In addition to the three systems, the expression of CB1 and ERα, two receptors of enormous importance in this matter, was studied.

Regarding CB1, the absence of significant effects induced by THC in the HF was a surprise—it is well established in the literature that it is through this receptor that THC elicits most of its effects [1,4,39]; therefore, we were expecting a different result. This may be partially explained by CB1 receptor internalization following activation by certain ligands, which is a process that can be dynamically regulated by cannabimimetic drugs [40,41]. In fact, some studies have found that some cannabinoid agonists have the ability to activate and internalize CB1 receptors (including in the hippocampus), which makes us think that THC might also have that specific effect. Despite that, the decrease in CB1 expression caused by EB is an interesting result, as it corroborates previous studies that described an interaction between estradiol and CB1 in a region-dependent manner [42]. However, determining the functional consequences of THC’s effects on CB1 is challenging due to the complex and tightly regulated processes of CB1 expression, trafficking, and degradation. This might be useful for comprehending the relationship between the cannabinoid system and the modulation of estradiol levels, considering this interaction has been described as a probable basis for the increased sensitivity observed in female *Cannabis* consumers [2,18].

Regarding ERα, we did not find significant effects of THC in any of the areas studied. There was also no significant effect found in the expression of this protein through EB administration, although some borderline statistics were found in the CA3 area (Oil vs. EB: *p* = 0.06; EB vs. THC-EB: *p* = 0.07), suggesting that despite the previous analysis regarding the minimum animal number required, in the present case, the sample number turned out to be small. However, since it was described in the literature that THC modulates estradiol release and its binding to the receptor [19], it is possible that the THC effects in the female endocrine system could primarily occur through those mechanisms rather than through direct effects on the ERα expression itself.

### 3.4. Limitations of the Study

Our study has a few limitations. We used a small sample size, which could have stopped us from achieving significant results regarding the relationship between THC and some of the proteins studied; however, the use of a larger sample size was not possible due to legal and logistical reasons. Notwithstanding previous power analysis and similar studies, the sample size became too small for the kind of data that we had, reducing the statistical power and our ability to see possible changes. This might explain the absence of effects detected in some of the proteins, particularly those whose relationship has been previously described in the literature (e.g., THC and CB1 or EB and ERα). Therefore, some of the results must be interpreted with caution.

The use of a single dose of THC might have also impacted our results. Although the use of more than one dose would be useful to study in more depth the relationship between THC and the proteins used, the aim of this study was not those actions of THC. In the same way, we could test a crescendo dose that would better mimic the social consumption of the drug. However, the aim of the present study was to analyze the effect of such a small dose of THC, mimicking the sparse smoke of a marijuana cigarette for pain or recreationally, and the short time frame of half a month would relate to about 9 months in human age [43]. In this way, we believe that longer time frames and other doses and schedules of administration for THC would be a good way to go forward in this field.

The timing of the tissue collection might have altered our results. According to the literature, THC has an initial half-life of approximately 6 min, with a terminal half-life of approximately 22 h [44]. The animals were sacrificed 6 h after the last THC injection, which is well beyond the initial half-life window, which might have camouflaged the acute effects of THC administration. However, we should point out that we studied animals that received daily injections of THC for 18 days, which might have been useful for detecting sub-chronic alterations.

It would have also been interesting to conduct behavioral studies regarding memory and attention before the rats were sacrificed—this was not the scope of this research in particular, considering this was conducted in a previous paper by some of the authors [34].

## 4. Materials and Methods

### 4.1. Animals and Experimental Design

This study involved twenty young-adult female Wistar rats (*Rattus norvegicus*), with five animals assigned to each experimental group. The subjects were sourced from the animal facility at the Faculty of Medicine, University of Porto (FMUP). Sample size determination was based on a prior power analysis (*n* = 5, power of 0.08, and type I error rate of 0.05) and in accordance with the legal determinations regarding replacement, reduction, and refinement that demand the usage of the minimum possible number of animals (Portuguese Act 113/13). The rats were kept in a controlled environment with a 12 h light/dark cycle, an ambient temperature of 21 ± 1 °C, relative humidity of 45 ± 5%, and free access to a standard solid diet and water. Regular monitoring included food and water intake, as well as body weight. Reproductive viability was confirmed through vaginal lavage, with only those exhibiting at least four complete estrous cycles being included in the study. All the procedures adhered to the European Communities Council Directive 2010/63/EU and Portuguese Act 113/13 and were approved by FMUP’s ethical review board (ORBEA).

Following a three-week acclimatization period, eight-week-old rats were anesthetized with ketamine (4 mg/kg) and medetomidine (0.05 mg/kg) before undergoing gonadectomy. Some of the rats received atipamezole (1 mg/kg) to counteract anesthetic effects, which was chosen because of its short-lasting action (two to three hours), which is less than the duration of the recovery phase. No antibiotic treatment was needed after surgery. A two-week recovery phase preceded experimental treatments. Two compounds, THC and estradiol benzoate (EB), were dissolved in 0.1 mL sesame oil and administered subcutaneously at 14:00 h; THC was administered every day, while EB was administered once every 4 days. The time of drug administration was chosen because that is the schedule that best mimics the physiological surge of estradiol [45]; THC was given at the same time to avoid extensive manipulation and distress to the animals. Half of the subjects received daily THC injections (1 mg/kg) throughout the study. This subcutaneous THC dose was selected for its translational relevance to both recreational and therapeutic human exposure and is consistent with previous reports [46]. Five days after THC administration began, the rats were further divided into two groups: one receiving sesame oil injections (Oil and THC groups) and the other receiving 10 μg of EB (EB and THC-EB groups) every four days from day six onward, spanning three hormonal cycles. The hormone doses were proven to mimic the physiological hormone values of female rats, as discussed in previous studies by the authors [47,48].

At the study’s conclusion (day 18, counted from the first injection of THC), the rats were euthanized thirty hours after the last EB administration and six hours after the last THC administration via intracardiac perfusion with 4% paraformaldehyde; their brains and uteri were extracted, with the uterine weight assessed to confirm hormone exposure. The schedule of animal sacrifice took place during the first third of the night, a moment when the estrogenic action is of relevance for female neuroendocrinology [34]. Serial 40 μm thick coronal sections were obtained from the brains and kept in storage boxes, each with twenty-four compartments, and preserved in Olmos solution [30% (*w*/*v*) sucrose, 30% (*v*/*v*) ethylene glycol, and 1% (*w*/*v*) polyvinylpyrrolidone-40 in phosphate-buffered saline (PBS)] at −20 °C until further analysis.

### 4.2. Immunohistochemistry

In order to analyze the expression of the various proteins mentioned above (i.e., CHRNA7, VAChT, VGLUT, GABRA, and GAD), individual sets of brain sections, comprising 1/12 sections of the entire dorsal HF, were processed per antibody. The sections were initially blocked for one hour in a solution containing 5% normal horse serum in PBS, followed by a 72-h incubation at 4 °C with the primary antibodies at predetermined dilutions, as detailed below (Table 1). Afterwards, the sections were washed in PBS and mounted on glass slides coded for the identification of each animal. The secondary antibody (Table 1) incubation proceeded on the glass slides and occurred for two hours at room temperature under dark conditions. Between each step, the sections were rinsed with PBS. Finally, coverslips were applied using FluorSave (Sigma-Aldrich, Merck Life Science S.L.U. sucursal em Portugal, Algés, Portugal) supplemented with DAPI (Thermo Fisher Scientific, Biogen Científica, Madrid, Spain) at a 1:100 dilution.

### 4.3. Fluorescence Quantification

**Figure 6:**
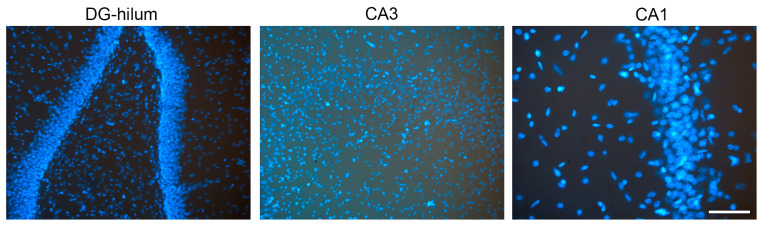
Examples of HF images obtained to analyze different areas and protein expression: DG-hilum, CA3, and CA1. Bar = 200 μm.

**Figure 7:**
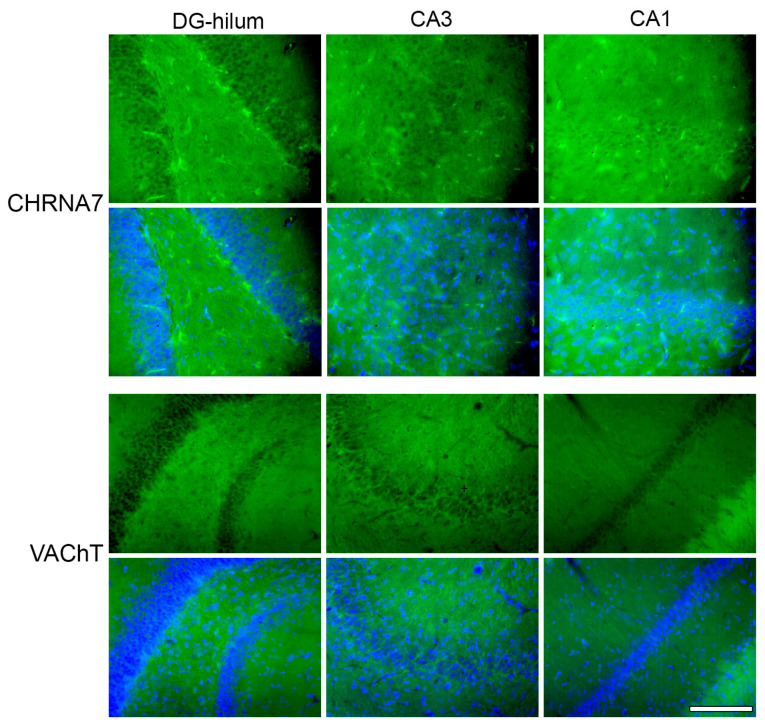
Representative photomicrographs of immunohistochemical staining of CHRNA7 and VAChT in all three areas analyzed: DG-hilum, CA3, and CA1. Every upper line shows an image stained for the specific antibody, and the lower line shows a merge with the DAPI image in order to better identify the areas. Bar = 200 μm.

**Figure 8:**
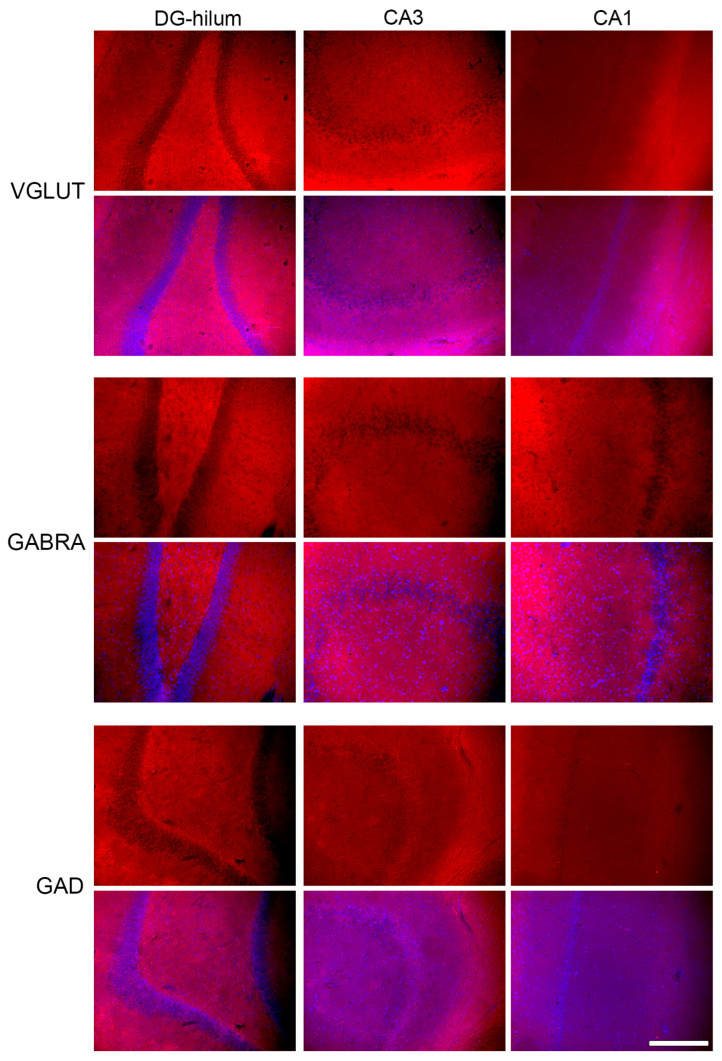
Representative photomicrographs of immunohistochemical staining of VGLUT, GABRA, and GAD in all three areas analyzed: DG-hilum, CA3, and CA1. Every upper line shows an image stained for the specific antibody, and the lower line shows a merge with the DAPI image in order to better identify the areas. Bar = 200 μm.

**Figure 9:**
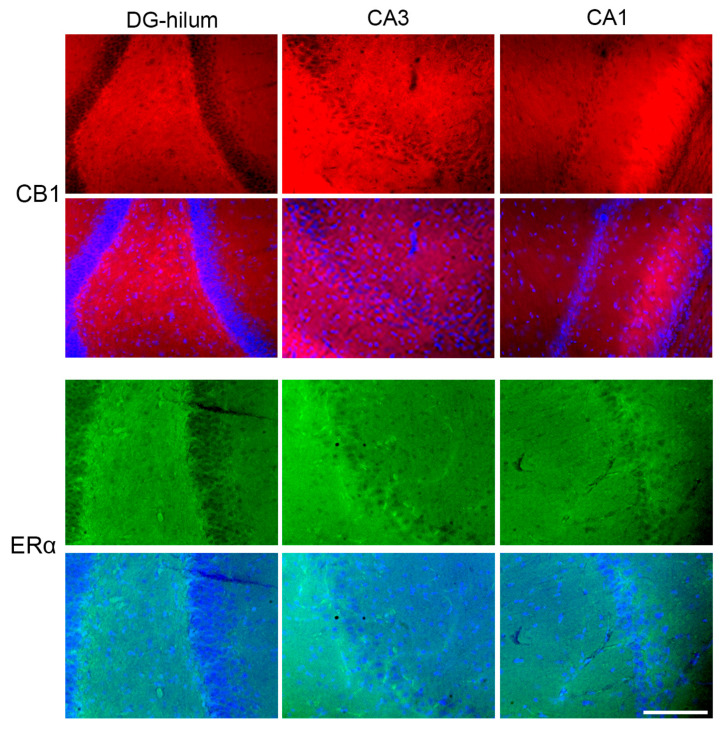
Representative photomicrographs of immunohistochemical staining of CB1 and ERα in all three areas analyzed: DG-hilum, CA3, and CA1. Every upper line shows an image stained for the specific antibody, and the lower line shows a merge with the DAPI image in order to better identify the areas. Bar = 200 μm.

Fluorescence imaging was conducted using a Leica DC 300F color video camera (Leica, Wetzlar, Germany) connected to a Leica DMR light microscope (Leica, Wetzlar, Germany), utilizing a 20×/0.40 objective lens (NPLAN PH1). The dorsal HF was identified alongside its rostrocaudal extent for each antibody. On average, four to five HF sections per rat were analyzed, with each section spaced at 480 μm. The selected area corresponded to the microscope camera’s field of view (97,655.482 μm^2^), capturing images of the granular layer and hilus of the DG, along with the inner and outer molecular and pyramidal layers of CA1 and CA3 (Figure 6). The image filename presented the code of the slide (corresponding to the animal)—the area analyzed—the filter used (DAPI; λ_488; λ_568)—index value. All areas were assessed separately. Consistent settings for the exposure time, gain, and offsets were applied to all image acquisitions. Optic density (OD) measurements of CHRNA7 and VAChT (Figure 7); VGLUT, GABRA and GAD (Figure 8); CB1 and ERα (Figure 9) expression were performed using the “Measure” tool of the ImageJ software program (version 2.14.0/1.54f; NIH, Bethesda, MD, USA). Briefly, FIJI software has the possibility to build a macro to create a script that automatically opens each image, allows the user to adjust the ROI if needed and select and separate the hilum ROI from the cellular layer of the DG, and measure the optic intensity in the ROI area. The results of the FIJI measurements presented the value for the ROI “Area” and for the “Integrated Density” in the same area for each image. Because the returned results file (.cvs) presents the same filename as the corresponding image, the results are identified in the same way. Data processing in Excel allows the separation of information regarding the brain area and filter used, and the ID code for each animal, which is then decoded to the corresponding treatment group. Finally, the sample information and data are used to calculate the OD by dividing the “Integrated Density” by the area of each image that corresponds to each rat sample.

### 4.4. Statistical Analysis

Data analysis was conducted using JASP Software (version 0.19.3.0; JASP Team, Amsterdam, The Netherlands). The normality of the distribution was assessed using the Shapiro–Wilk test. A one-way ANOVA was performed with a two-sided alpha of 0.05 and a power of 0.80 to compare the experimental groups. Post-hoc multiple comparisons were carried out using Tukey’s tests.

## 5. Conclusions

We showed that both THC and EB can modulate neurotransmission in the HF, which can have significant implications for cognitive functions, particularly memory and learning. This is consistent with previous research, as referenced above. Besides the individual effects of both compounds, we were also able to demonstrate an interaction between THC and EB, which might help explain the existence of sex differences in *Cannabis* consumption. This knowledge can be applied in the medical and pharmaceutical fields to develop new drugs for CNS-related diseases.

Further studies are needed to better correlate neurotransmission changes and cognitive effects, including changes in behavioral responses. It might also be interesting to investigate the effects of higher doses of THC and/or longer interventions to understand the differences (if any) between acute and chronic THC administration. The reversibility of the brain effects of THC would also be an important subject of investigation, especially with the increase in the consumption of *Cannabis* by the population.

## Figures and Tables

**Table 1:** Antibody detailed description.

Antibody	Antibody Registry ID	Dilution	Supplier
Primary antibodies			
Rabbit anti-CHRNA7 Polyclonal Antibody (PA5-116644)	AB_2901275	1:500	Thermo Fisher Scientific (Biogen Científica, Madrid, Spain)
Goat Anti-VAChT Polyclonal Antibody (AG260)	AB_10000324	1:500	Merck Millipore (Algés, Portugal)
Mouse anti-VGLUT2 Monoclonal Antibody (MAB5504)	AB_2187552	1:1000	Merck Millipore (Algés, Portugal)
Mouse anti-GABRA5 Monoclonal Antibody (MA5-27700)	AB_2735193	1:500	Thermo Fisher Scientific (Biogen Científica, Madrid, Spain)
Mouse anti-GAD67 Monoclonal Antibody (MAB5406)	AB_2278725	1:2000	Merck Millipore (Algés, Portugal)
Rabbit anti-Cannabinoid Receptor 1 Polyclonal Antibody (ab23703)	AB_447623	1:500	Merck Millipore (Algés, Portugal)
Rabbit anti-ERα Polyclonal Antibody (sc-542)	AB_631470	1:1000	Merck Millipore (Algés, Portugal)
Secondary antibodies			
DyLight 488 Goat anti-rabbit (DI-1488)	AB_2336402	1:1000	Vector Laboratories (Oxford, UK)
Alexa Fluor™ 488 Donkey anti-Goat IgG (A-11055)	AB_2534102	1:1000	Thermo Fisher Scientific (Biogen Científica, Madrid, Spain)
Texas Red Horse anti-mouse (TI-2000)	AB_2336178	1:1000	Vector Laboratories (Oxford, UK)

## Data Availability

The data that support the findings of this study are available from the corresponding author upon reasonable request.

## Abbreviations

The following abbreviations are used in this manuscript:

CA	*Cornu ammonis*
CB1	Cannabinoid receptor type 1
CB2	Cannabinoid receptor type 2
CHRNA7	Cholinergic receptor alpha 7 subunit
CNS	Central nervous system
DG	Dentate gyrus
EB	Estradiol benzoate
EBα	Estradiol receptor alpha
EC	Entorhinal cortex
GABA	Gamma-aminobutyric acid
GABRA	Gamma-aminobutyric acid type A receptor
GAD	Glutamic acid decarboxylase
HF	Hippocampal formation
OD	Optic density
PBS	Phosphate-buffered saline
SC	Subicular complex
SEM	Standard error of the mean
THC	Delta-9-tetrahydrocannabinol
VAChT	Vesicular acetylcholine transporter
VGLUT	Vesicular glutamate transporter
